# Differences in Muscle Strength in Parkinsonian Patients Affected on the Right and Left Side

**DOI:** 10.1371/journal.pone.0121251

**Published:** 2015-03-25

**Authors:** Giuseppe Frazzitta, Davide Ferrazzoli, Roberto Maestri, Roberta Rovescala, Gabriele Guaglio, Rossana Bera, Daniele Volpe, Gianni Pezzoli

**Affiliations:** 1 Department of Parkinson Disease and Brain Injury Rehabilitation, “Moriggia-Pelascini” Hospital, Gravedona ed Uniti, Italy; 2 Department of Biomedical Engineering, Scientific Institute of Montescano, S. Maugeri Foundation, Istituto di Ricovero e Cura a Carattere Scientifico (IRCCS), Montescano, Italy; 3 Department of Neurorehabilitation, Scientific Institute of Montescano, S. Maugeri Foundation, Istituto di Ricovero e Cura a Carattere Scientifico (IRCCS), Montescano, Italy; 4 Department of Physical Medicine and Rehabilitation, “S. Raffaele Arcangelo Fatebenefratelli” Hospital, Venezia, Italy; 5 Parkinson Institute, Istituti Clinici di Perfezionamento, Milano, Italy; 6 Fondazione Europea Ricerca Biomedica (FERB), “S.Isidoro” Hospital, Trescore Balneario, Italy; University of Tuebingen, GERMANY

## Abstract

**Background:**

Muscular weakness is a frequent cause of instability that contributes to falls in Parkinson’s disease (PD). Isokinetic dynamometry is a method of muscle assessment useful to measure the muscular strength giving a quantification of the weakness, but only few studies about isokinetic assessment were performed in PD. The aims of the study were to evaluate the muscle strength in PD and to investigate the differences in patients affected on the right and left side.

**Methods:**

Knee flexor and extensor muscles strength was assessed using an isokinetic dynamometer in 25 patients in stage 3 H&Y and in 15 healthy controls. Subjects were tested in both legs at three fixed angular velocities: 90°/s, 120°/s, 180°/s.

**Results:**

Considering the whole population of Parkinsonians, no difference in strength was observed with respect to controls. Considering the side, patients affected on the right side showed a clear tendency to be weaker than patients affected on the left side and controls.

**Conclusions:**

PD patients affected on the right side, but not those affected on the left side, had a reduction in muscle strength as compared to controls. We postulate a central origin deficit in muscle strength in PD. It is known that dopamine transporter binding is more severely reduced in the left posterior putamen and our results suggest that the control of the muscle strength in PD is linked to the right–left hemispheric asymmetry of the functional organization of basal ganglia and with their connections to cortical motor and pre-motor areas.

## Introduction

Parkinson’s disease (PD) is a neurodegenerative disorder characterized by different motor symptoms (rigidity, akinesia, tremor, impairment of balance and gait). Postural instability and falls are the most relevant problems in PD and they have a dramatic impact on patients’ quality of life [1]. Since muscular deficiency is a frequent cause of instability and unbalance in elderly people, proper muscular assessment is important for preservation of joint stability and for preventing falls and disability. Isokinetic dynamometry is one of the most accurate methods of muscle assessment [2,3]. An isokinetic muscle action is defined by its performance at a constant speed. The laboratory measurement of isokinetic strength provides torque measurements throughout the active range of motion during a maximal effort. Torque is the tendency of a force to rotate an object about an axis and is produced and recorded from the angular motion. Consequently, the peak torque is an index of the muscular strength. Isokinetic testing was introduced as a quantitative measurement of both static and dynamic muscular contraction in which all variables involved (resistance, limb velocity, and joint position) are under control. This is because isokinetic muscle testing allows objective, valid, and reliable measurement of the force produced by skeletal muscle during exercise at constant velocity and, accommodating resistance, it is appropriate for measuring muscle strength and muscle endurance across the disability spectrum [4–6]. Along with impaired proprioception, visual dysfunctions and reduced base of support, the reduction of muscle strength seems to be a cause of postural instability and falls in PD patients [11].

However, there are only a few published studies about isokinetic assessment for the flexor and extensor muscles of the knee in PD [7, 10–13]. The weakness in PD has been reported to be present bilaterally, to be related to the disease progression and increasing with performance velocity [10]. Conversely, other studies confirmed a decrease in isokinetic muscle strength in PD, but they did not find a relationship between decreased muscle strength and movement velocity [7,11]. Although a reduced rate of muscle force production has been documented in PD [12], evidences supporting a reduced magnitude of force in PD patients are less definitive [13]. This result might be influenced by the specific muscle tested, by the severity of the disease, by the type of muscle action, or by the specific strength analysed [13]. However, these studies showed that the isokinetic evaluation of muscle strength is important for the assessment of severity and falls in PD [7,10]: *Corcos et al* demonstrated that there is an asymmetric distribution of weakness in PD and that muscle strength and muscle relaxation correlate with changes in clinical status [12]. Specifically, it has been suggested that reduced strength in individuals with mild PD contributes to the difficulty of Parkinsonian patients to perform postural changes (i.e. arise from a chair) [13]. Despite these suggestions, the problem of weakness in PD has not been clarified yet, as well as the possible link between weakness asymmetry, handedness and affected side.

The aims of the present study were: 1) to evaluate the muscle strength in Parkinsonian patients in the medium stage of disease, using an isokinetic dynamometer, comparing the results with a control group of healthy people of similar age and 2) to investigate the existence and to assess the differences in muscle strength in Parkinsonian patients predominately affected on the right and left side.

## Materials and Methods

### 1. Participants

Patients with a diagnosis of “clinically probable” idiopathic Parkinson’s disease according to Gelb et al [8], were screened by a neurologist specialized in movement disorders. Inclusion criteria were: i) Hoehn-Yahr stage 3, ii) stable pharmacological treatment in the last eight weeks, iii) mini-mental state examination score (MMSE) > 25. Patients were excluded if they had neurological conditions other than idiopathic Parkinson’s disease, cardiovascular disorders, hip, knee or ankle dysfunctions or psychiatric disorders that could interfere with the performance.

Screening was stopped when twenty-five patients were enrolled. Fifteen healthy subjects matched for age were enrolled in the control group. All patients and controls were right-handed on the basis of the anamnesis and clinical assessment. Patients were defined as left side or right side affected considering the anamnesis and on the basis of clinical neurological examination.

Muscle strength evaluation was performed in the morning at 9 A.M for both groups. PD patients were evaluated in ON state.

The study was approved by the local Scientific Committee and Institutional Review Board (Fondazione S. Maugeri, IRCCS, Istituto Scientifico di Montescano). Written informed consent was obtained from all patients before participation.

### 2. Equipment

Knee flexor and extensor muscles strength was assessed using an isokinetic dynamometer (Cybex Norm) equipped with a computerized system allowing arcs of movement at the desired constant angular velocity. Muscle strength was measured in both lower limbs with the subjects in the sitting position with hip flexed at 90°. The trunk and the thigh to be tested were tightly secured by velcro-straps to the experimental chair. The mechanical lever arm was aligned to the axis of rotation of the knee. To account for the influence of the gravity effect torque, data were corrected according to the weight of the subject's lower limb at 45° (it refers to 45° of flexion, i.e 135 of extension). Subjects were tested at three fixed angular velocities: 90°/s (degree/second), 120°/s, 180°/s, likewise previously performed [7]. The subjects began each isokinetic contraction with the knee flexed at 90° and continued through the full range of motion. On the test day, the subjects performed several warm-up trials at submaximal levels to ensure a thorough familiarization with the equipment. They were then asked to complete five maximal contractions at each angular velocity in a randomized order. Only the best value was retained for further statistical analysis. All participants were verbally encouraged to perform each test as maximally as possible. Visual feedback during the test was also provided. The same investigator conducted the tests for all subjects. The impact artefact was not included in the measurement of maximal torque. A recovery of 1 minute was allowed between each maximal contraction, to minimize fatigue.

Isokinetic knee extensor and flexor strength (peak torque) at 90°/s, 120°/s and 180°/s was measured in both legs.

### 3. Statistical analysis

Descriptive statistics are reported as median (lower quartile—upper quartile) and mean±SD for, respectively, non-normally and normally distributed data.

Comparisons for categorical variables were carried out by the Chi-square test or Fisher exact test, when appropriate. Between groups comparisons were carried out by Mann-Whitney U-test for two groups and by the Kruskal Wallis test when three groups were considered (patients predominately affected on the right side, patients predominately affected on the left side and controls). The level of significance was set at 0.05. In case of statistically significant difference between the three groups, providing evidence that at least one of the population medians differed from the others, pairwise comparisons were carried out to determine which specific population median could be considered significantly different from each other.

All analyses were carried out using the SAS/STAT statistical package, release 9.2 (SAS Institute Inc., Cary, NC, U.S.A.).

## Results

Demographic and clinical data for PD patients and healthy subjects are reported in Table 1.

**Table 1:** Demographic and clinical data of patients and controls.

**Variable**	**Patients**	**Controls**	**p-value**
**Age (yrs)**	67.9 ± 6.5	65.7 ± 8.3	0.321
**Left/Right side affected**	14/11		
**Sex (% male)**	65	47	0.335
**Height (cm)**	168 ± 7	170 ± 6	0.276
**Weight (Kg)**	69 ± 4	70 ± 6	0.314
**BMI (Kg/m2)**	24.7 ± 1.0	25.0 ± 0.8	0.435
**UPDRS III**	20 (18–25)		
**Daily Levodopa equivalent dose (mg)**	620 ± 285		
**MMSE**	29 (27–30)		
**Original Handedness (%)**	100 Right, 0 left	100 Right, 0 left	

*Abbreviations*: *BMI (Boby Mass Index); UPDRS (Unified Parkinson's Disease Rating Scale); MMSE (Mini Mental State Examination)*.

In Table 2 data for the two cohorts of PD patients (right affected and left affected) are compared.

**Table 2:** Comparison between right affected and left affected PD patients.

**Variable**	**Right side affected PD patients**	**Left side affected PD patients**	**p-value**
**Age**	69 ± 5	67 ± 8	0.3379
**Sex (% male)**	55	71	0.3827
**Height (cm)**	171 ± 7	172 ± 6	0.5876
**Weight (Kg)**	67 ± 11	70 ± 10	0.3850
**BMI (Kg/m2)**	22.7 ± 2.1	23.6 ± 2.3	0.3183
**UPDRS III**	23 (18–31)	20 (16–21)	0.3100
**Daily Levodopa equivalent dose (mg)**	626 ± 273	615 ± 305	0.9213
**MMSE**	29 (27–30)	29 (28–30)	0.8977

*Abbreviations*: *BMI (Boby Mass Index); UPDRS (Unified Parkinson's Disease Rating Scale); MMSE (Mini Mental State Examination); PD (Parkinson’s disease)*.

No differences were observed between groups. Extensor and flexor strength at 90°/s, 120°/s and 180°/s in left and right knee for PD patients and healthy subjects are reported in Table 3.

**Table 3:** Extensor and flexor muscle strength (peak torque) at 90°/s, 120°/s and 180°/s in Patients and Controls.

**Variable**	**Patients**	**Controls**	**p-value**
**Right knee extensor peak torque at 90°/s**	66.0 (40.3–81.5)	66.0 (43.8–100.5)	0.665
**Right knee extensor peak torque at 120°/s**	45.0 (29.5–66.5)	42.0 (34.3–85.8)	0.476
**Right knee extensor peak torque at 180°/s**	35.0 (26.0–55.5)	41.0 (30.0–73.3)	0.356
**Left knee extensor peak torque at 90°/s**	56.0 (40.8–76.3)	75.0 (46.5–94.3)	0.204
**Left knee extensor peak torque at 120°/s**	43.0 (25.5–56.5)	49.0 (38.3–85.3)	0.371
**Left knee extensor peak torque at 180°/s**	34.0 (19.8–47.8)	39.0 (31.8–71.2)	0.180
**Right knee flexor peak torque at 90°/s**	28.0 (22.8–42.0)	43.0 (25.8–78.8)	0.198
**Right knee flexor peak torque at 120°/s**	24.0 (18.8–30.3)	42.0 (23.3–68.7)	0.067
**Right knee flexor peak torque at 180°/s**	20.0 (18.0–31.0)	37.0 (19.3–50.8)	0.146
**Left knee flexor peak torque at 90°/s**	28.0 (20.0–35.8)	45.0 (27.0–65.3)	0.048
**Left knee flexor peak torque at 120°/s**	23.0 (19.8–34.0)	39.0 (21.8–60.0)	0.093
**Left knee flexor peak torque at 180°/s**	23.0 (17.5–31.0)	39.0 (19.0–53.8)	0.102

Peak torque values are expressed in Newton Metre (N·m).

Considering the whole population of PD patients, no difference in strength was observed with respect to healthy subjects, with the exception of a significant (p = 0.048) difference in flexor strength at 90°/s, left knee. Considering the two different groups of PD patients (i.e. those predominately affected on the right side and those predominately affected on the left side), results of the comparison of strength with control subjects are given in Table 4.

**Table 4:** Extensor and flexor strength (peak torque) at 90°/s, 120°/s and 180°/s in PD patients predominately affected on the left and on the right side (Left PD and Right PD, respectively), and controls.

**Variable**	**Left PD (N = 14)**	**Right PD (N = 11)**	**Controls (N = 15)**	**p-value**
**Right knee extensor peak torque at 90°/s**	71.5 (50.0–99.0)	50.0 (35.0–68.8)	66.0 (43.8–100.5)	0.131
**Right knee extensor peak torque at 120°/s**	54.0 (34.0–85.0)	35.0 (24.0–52.8)	42.0 (34.3–85.8)	0.100
**Right knee extensor peak torque at 180°/s**	43.5 (33.0–73.0)	28.0 (17.0–47.0)	41.0 (30.0–73.3)	0.077
**Left knee extensor peak torque at 90°/s**	62.0 (52.0–111.0)	50.0 (21.8–63.5)	75.0 (46.5–94.3)	0.032
**Left knee extensor peak torque at 120°/s**	48.5 (28.0–100.0)	43.0 (14.0–52.0)	49.0 (38.3–85.3)	0.134
**Left knee extensor peak torque at 180°/s**	34.5 (26.0–77.0)	34.0 (16.0–41.3)	39.0 (31.8–71.2)	0.135
**Right knee flexor peak torque at 90°/s**	38.0 (27.0–75.0)	23.0 (22.0–30.3)	43.0 (25.8–78.8)	0.023
**Right knee flexor peak torque at 120°/s**	24.0 (19.0–56.0)	20.0 (15.0–26.3)	42.0 (23.3–68.7)	0.070
**Right knee flexor peak torque at 180°/s**	21.5 (19.0–52.0)	20.0 (13.5–23.8)	37.0 (19.3–50.8)	0.148
**Left knee flexor peak torque at 90°/s**	31.0 (28.0–77.0)	20.0 (15.0–29.0)	45.0 (27.0–65.3)	0.004
**Left knee flexor peak torque at 120°/s**	28.0 (22.0–68.0)	22.0 (16.5–23.8)	39.0 (21.8–60.0)	0.042
**Left knee flexor peak torque at 180°/s**	24.0 (18.0–50.0)	22.0 (17.0–27.0)	39.0 (19.0–53.8)	0.108

Peak torque values are expressed in Newton Metre (N·m). P value is from the group comparison (Kruskal Wallis test).

In general, patients affected on the right side showed a clear tendency to be weaker than patients affected on the left side and controls, with statistically significant differences (Kruskal Wallis test) in left knee extensor peak torque at 90°/s, right knee flexor peak torque at 90°/s, left knee flexor peak torque at 90°/s and left knee flexor peak torque at 120°/s.

Considering these variables, we found no differences between left side affected patients and controls, while right side affected patients were significantly weaker than controls (p = 0.014, p = 0.019, p = 0.001 and p = 0.021 respectively) and significantly weaker than left side affected patients for left knee extensor peak torque at 90°/s, right knee flexor peak torque at 90°/s, left knee flexor peak torque at 90°/s (p = 0.037, p = 0.014, p = 0.015 respectively).

## Discussion and Conclusions

In contrast to previous studies [9], our study does not reveal any statistical differences in muscle strength values between PD patients and age-matched healthy subjects for all the variables considered. However, there is a trend indicating a greater strength in healthy subjects. In order to better understand the complex problem of weakness in PD, we compared right side affected PD patients with left side affected PD patients. In this case, we found that PD patients with the right side predominately affected show a reduction in muscle strength compared to controls and to PD patients with left side affected; whereas, in left side affected PD patients all recorded muscle strength parameters result comparable to controls. Therefore, for all measurements, there is a greater muscular strength in left side affected PD patients than in right side affected PD patients. The differences between the right and left sided cohort is especially present testing the muscle strength at 90°/s. We hypothesized that this difference is greater at this angular speed because 90°/s it is the starting point of the exercise and we know that the beginning of movement is dysfunctional in Parkinsonian patients.

It has been previously reported that PD patients have reduced muscle strength, decreased rate of force development, impaired ability to maintain constant force [16], and increased muscle coactivation during balance perturbation tasks [9]. The origin of weakness remains unclear: is it peripheral or central, intrinsic to the disease or is it a secondary phenomenon?

Peripheral changes have been reported in PD: muscles biopsies taken from the biceps brachii and tibialis anterior from persons with PD have shown increased type-I fibres and decreased type-II fibres, but it is not known whether these muscle structural rearrangements are attributed to the disease process or are secondary to reduced mobility [13–15]. It is well known that in many cases peripheral changes in nerve or muscle are unlikely to explain the weakness [9].

Moreover, diminished motor performance caused by weakness may be a primary sign of PD and may be explained by disturbed motor programming in basal ganglia [10].

Recently, Moreno Català and colleagues demonstrated that the reduced muscle strength of the leg extensor in PD patients could be highly accounted for by the increased antagonistic movement and the increased activation deficit of the agonist [16]. These authors postulated the existence of a central origin deficit in muscle strength: PD falling patients are not able to activate the right muscles for the task, suggesting an important role of the basal ganglia in optimizing muscle synergy patterns [16]. Furthermore, a decrease in muscle activation and motor unit behaviour alterations have been demonstrated in PD so that the discharge patterns of motor units are irregular and intermittent and antagonist muscles are abnormally coactivated [17].

Our results seem to indicate that muscle strength in PD patients is not a discriminating factor, but it tends to be reduced when compared to healthy subjects, contributing to weakness. Since PD patients with the right affected side, but not with the left one, have a reduction in muscle strength compared to controls, we postulated a central origin deficit in muscle strength in PD too. However, our data suggest that the origin of the central muscle strength deficit in PD is not only related to a problem of central recruitment and muscular activation, but it is intimately associated with the side of the disease: this may mean that this “central weakness” in PD is linked to a problem of intra-hemispheric dissociation.

PD has a unilateral onset, providing evidence of intra-hemispheric dissociation and an imbalance in the relative strengths of the right and left hemispheres [18]. Breaks in cortico-striato-thalamo-cortical and cortico-cortical circuitry have been invoked to explain the variety of motor and non-motor symptoms in Parkinsonian patients [19,20]. In particular, in PD there is evidence of intrahemispheric dissociations and imbalance between the right and left hemispheres, in relation to the side of onset of the disease. The asymmetrical motor symptoms of PD are associated with asymmetrical depletion of dopamine in the substantia nigra [21,22]. These changes in the substantia nigra lead to asymmetrical dysregulation of the striatum, with consequent asymmetrical dysfunction of neural circuits, including basal ganglia and cortical areas [23].

How could these considerations explain the differences in strength between PD patients with left or right side affected? Scherfler C. and colleagues have recently shown that dopamine transporter binding in Parkinsonian patients was more severely reduced in the left posterior putamen compared with the right one [24]. This appears consistent with other recent studies, showing greater proportions of right-handed PD patients with greater motor impairment of their right side compared with their left sided limbs [25,26].

Our results show that Parkinsonian patients affected on the right side, but not on the left side, have a reduction in muscle strength as compared to controls. This may mean that the strength deficit can be related with a hemispheric dominance.

Motor dominance of the left hemisphere is associated with increased vulnerability of the left dopaminergic nigro-striatal projections of right-handed patients with PD [24]. However, there was still a considerable number of right-handed PD patients with lower dopamine transporter binding of their right putamen [24]. This clearly argues against hemispheric dominance as the only factor determining asymmetric nigrostriatal dysfunction in PD [24]. In line with this, all our patients are right-handers. They are independently affected on the right or on the left side, but those with the right side affected are weaker than those with the left side affected.

These findings suggest that the disrupted mechanism of the central muscle strength control in PD is intrinsically linked to the right–left hemispheric asymmetry of the functional organization of basal ganglia motor circuits: this is probably related to the left hemispheric predominance of nigrostriatal dysfunction [24] and with the diseased basal ganglia connections to motor and pre-motor areas of the cerebral cortex.

The relatively small sample size constitutes the main limitation of our study. Furthermore, no left-handed subjects were found among patients and controls. Further studies are needed to better understand the mechanisms underlying asymmetric motor features in PD.
